# Bmal1 knockdown in the left stellate ganglion inhibits neural activity and prevents ventricular arrhythmias after myocardial ischemia

**DOI:** 10.3389/fcvm.2022.937608

**Published:** 2022-09-29

**Authors:** Zhongyang Yu, Zhihao Liu, Liying Jiao, Song Zhang, Liqing Nie, Yueyi Wang, Liping Zhou, Yuhong Wang, Zhihao Liu, Zihan Liu, Xiao Xu, Zeyan Li, Yuyang Zhou, Huixin Zhou, Rui Li, Chen Peng, Lilei Yu, Hong Jiang

**Affiliations:** ^1^Department of Cardiology, Renmin Hospital of Wuhan University, Wuhan, China; ^2^Hubei Key Laboratory of Autonomic Nervous System Modulation, Wuhan, China; ^3^Cardiac Autonomic Nervous System Research Center of Wuhan University, Wuhan, China; ^4^Taikang Center for Life and Medical Sciences, Wuhan University, Wuhan, China; ^5^Cardiovascular Research Institute, Wuhan University, Wuhan, China; ^6^Hubei Key Laboratory of Cardiology, Wuhan, China

**Keywords:** Bmal1, left stellate ganglion, myocardial ischemia, ventricular arrhythmias, transcriptomics

## Abstract

**Objectives:**

The neural activity of the left stellate ganglion (LSG) is closely related to the occurrence of ventricular arrhythmias (VAs). Bmal1 modulates genes associated with neural activity in the central nervous system. However, few studies indicated the role of Bmal1 in the LSG and the subsequent effect on the heart. Therefore, we aimed to investigate the influence of Bmal1 knockdown in the LSG on its neural activity and cardiac electrophysiology and to explore the mechanisms.

**Materials and methods:**

We used adeno-associated virus (AAV) to knock down Bmal1 in the LSG. Male beagles were randomized into the Bmal1 knockdown group and the control group. After 4 weeks of injection, the LSG function, neural activity, left ventricular effective refractory period (ERP), and action potential duration (APD) were measured. Electrocardiography for 1 h was recorded for VAs analysis after myocardial ischemia. Nerve growth factor (NGF) and c-fos in the LSG were quantified by immunofluorescence. Transcriptomic analysis was performed to assess the gene expression in the LSG.

**Results:**

Bmal1 was sufficiently knocked down by AAV. Compared with the control group, heart rate variability (HRV) in the knockdown group was altered. Bmal1 knockdown inhibited neural activity and function of LSG. It also prolonged ERP as well as APD90. Ischemia-induced VAs were significantly reduced. Nerve growth factor (NGF) and c-fos in the LSG were reduced. Bmal1 knockdown led to the expression changes of genes associated with neural activity in the LSG.

**Conclusion:**

Bmal1 knockdown in the LSG suppresses neural activity and prevents ventricular arrhythmias after myocardial ischemia.

## Introduction

Cardiovascular disease (CVDs) is the major reason for mortality each year. Among CVDs, malignant ventricular arrhythmias (VAs) contribute to sudden death in patients with myocardial ischemia. It is well recognized that the neural activity of the cardiac sympathetic nervous system (SNS), including the left stellate ganglion (LSG), plays an important role in the occurrence of VAs (1).

A thought-provoking phenomenon has been widely observed in clinical and animal experiments: the occurrence of VAs fluctuates throughout the day and night; in other words, the occurrence of VAs has circadian rhythms (2). Another fact is that it has been proven in clinical practice that the neural activity of the SNS has an obvious oscillation during the day, and abnormal SNS discharges could lead to vulnerability to CVDs (3–5). All of these clues indicate that clock genes may be involved in the development of CVDs.

Bmal1 has been proven to be a crucial clock gene, which encodes transcription factors modulating downstream genes by binding to their enhancer box elements (E-boxes) (6, 7). These genes are related to a variety of processes, such as metabolism, growth, and even neural activity (8–10). In the central nervous system, Bmal1 concentrations in hypothalamic cells could affect urinary catecholamine concentrations by regulating catecholamine metabolism (11). At the same time, Bmal1 plays an important role in the neurons of the suprachiasmatic nucleus (SCN) (12). Moreover, deletion of Bmal1 in SCN astrocytes altered cognitive function by regulating γ-aminobutyric acid (GABA) reporters signaling (13). However, we still have no knowledge about the role of Bmal1 in the peripheral nervous system and the changes in cardiac electrophysiology caused by Bmal1 deficiency in the cardiac autonomic nervous system, especially the LSG.

Thus, in our study, we knocked down the expression of Bmal1 in the LSG to explore its influence on neural activity, and the occurrence of VAs after myocardial ischemia, and elucidate its molecular mechanisms.

## Materials and methods

### Animal preparation

Fourteen adult male beagles with about 10–12 kg body weight were selected. The Center of Experimental Animals in the Medical College of Wuhan University supported animals in our experiments. This study was performed in accordance with the recommendations in the Guide for the Care and Use of Laboratory Animals of the National Institutes of Health. The committee on the Ethics of Animal Experiments of Wuhan University approved the protocol (WDRM 20180507). The details of the experiments including the methods of anesthetization, air control, maintenance of body fluid volume and core body temperature, and body surface electrocardiography (ECG) recording were as previously described (14).

### Study protocol

**FIGURE 1:**
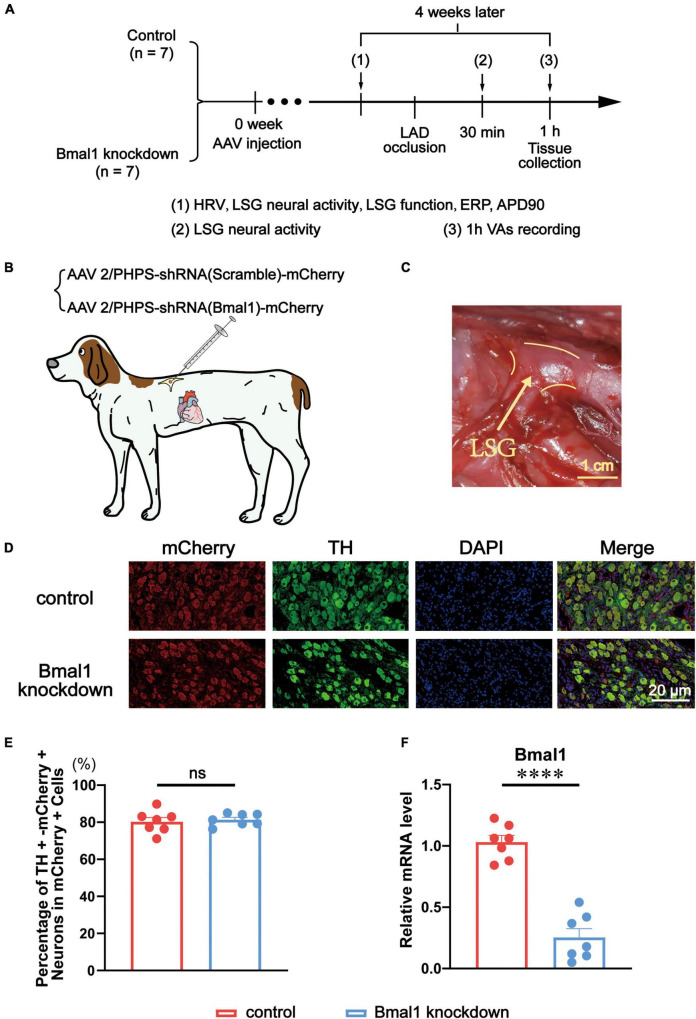
Evaluation of Bmal1 knockdown in the LSG. **(A)** Flowchart of the experiment. **(B)** Schematic diagram of virus microinjection in canines’ LSG. **(C)** The anatomical location of the LSG. **(D,E)** The percentage of TH + mCherry + neurons in mCherry + cells. **(F)** The expression of Bmal1 was analyzed by quantitative RT-PCR. *n* = 7 for each group. ^*⁣*⁣**^p < 0.0001. ns, *p* > 0.05. AAV, adeno-associated virus; HRV, heart rate variability; LSG, left stellate ganglion; ERP, effective refractory period; APD, action potential duration; LAD, left anterior descending coronary arteries; VAs, ventricular arrhythmias; TH, tyrosine hydroxylase; DAPI, 4,6-diamidino-2-phenylindole.

Fourteen adult male beagles were randomly divided into two groups: the control group (*n* = 7) and the Bmal1 knockdown group (*n* = 7). Four weeks after the microinjection of adeno-associated virus (AAV) as the tool to intervene in the expression of Bmal1, we measured the heart rate variability (HRV), LSG neural activity, LSG function, left ventricular effective refractory period (ERP), and left ventricular action potential duration (APD). Then, we occluded the left anterior descending coronary arteries (LAD) to establish a model of myocardial ischemia. Thirty minutes later, we measured the LSG neural activity again. At the same time, we recorded VAs by ECG for 1 h after myocardial ischemia, and finally, we collected the tissues (Figure 1A).

### Microinjection of adeno-associated virus

In the Bmal1 knockdown group, AAV (AAV2/PHPS-hSyn-mCherry-5′miR-30a-shRNA (Bmal1)-3′miR-30a-WPREs) was used as a genome-editing tool delivering a short hairpin RNA (shRNA) against canine Bmal1 into the LSG. AAV (AAV2/PHPS-mCherry-hSyn-5′miR-30a-shRNA (scramble)-3’miR-30a-WPREs) was used as a control (Figures 1B,C). We purchased the virus from Brain VTA (Wuhan, China). A left thoracotomy at the third intercostal space was performed under anesthesia. The LSG was carefully exposed, and a solution containing the virus was injected into two sites with a microsyringe at a flow rate of 2 μl per min. After the injection, the chest was closed in layers, and antibiotics (penicillin sodium) were administered for 3 days after surgery.

### Analysis of heart rate variability

Heart rate variability (HRV) is a classical indicator to evaluate sympathetic and vagal tone. It was measured by the Power Lab System (8/35, AD Instruments, Bella Vista, Australia). In the experiments, we fixed electrodes on the surface of all four legs of each canine. Then, we connected the limb leads, recorded their body surface ECG for at least 10 min, and analyzed the HRV. We analyzed three power spectral indices: the normalized units of low-frequency power (LFnu), the normalized units of high-frequency power (HFnu), and the ratio of LF/HF with the software in the system [LFnu = LF/(LF + HF) and HFnu = HF/(LF + HF)] (15). The unit of LF and HF is “ms^2.”

### Analysis of the left stellate ganglion function

We defined LSG function as the change in maximal systolic blood pressure with electrical stimulation (frequency: 20 Hz, pulse width: 0.1 ms). Then, we applied stimulation at four incremental voltage levels (level 1 was defined from 1 to 4 V; level 2 was defined from 5 to 7 V; level 3 was defined from 7.5 to 10 V, and; level 4 was defined from 10 to 15 V) by a Grass-S88 stimulator (Astro-Med, West Warwick, Rhode Island). The details of the operation were as previously described (14).

### Analysis of the left stellate ganglion neural activity

We recorded the neural activity of LSG for at least 5 min before MI and after MI. The neural activity was described in two aspects: amplitude and frequency of neural firing. To describe the influence of MI on activating LSG, we analyzed the alteration of neural activity before MI and after MI. Details of experiments including microelectrode embedding into LSG, equipment used to record neural activity, and data analysis were as previously described (14).

### Analysis of the ventricular effective refractory period and monophasic action potential duration

As cardiac electrophysiological parameters before MI, ERP, and APD of three sites at the left ventricular apex (LVA), the left ventricular base (LVB), and the mid left ventricle (LVM) in the two groups were recorded and 90% repolarization duration (APD90) was analyzed. The details of the experiments including using catheters, application of a pacing train, and data analysis were as previously described (14).

### Establishments of myocardial ischemia and analysis of myocardial ischemia-induced ventricular arrhythmias

**FIGURE 2:**
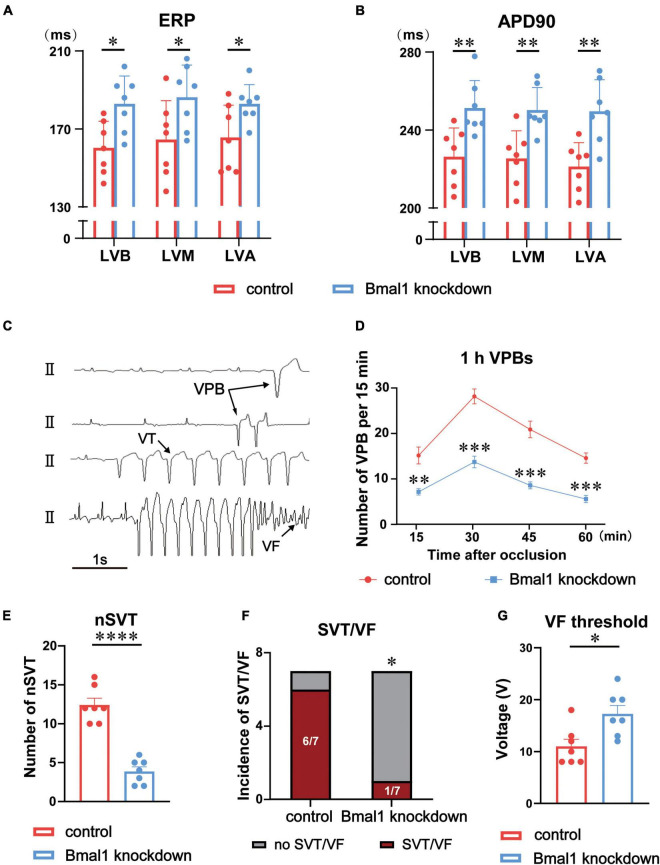
Effects of Bmal1 knockdown in the LSG on ERP and VAs after ischemia. **(A)** Left ventricular ERP and **(B)** APD90 in the two groups at three sites: LVB, LVM, and LVA. **(C)** Patterns of VAs. Quantitative analysis of the occurrence of ischemia-induced VAs including **(D)** 1 h VPBs, **(E)** nSVT, and **(F)** the incidence of SVT and VF. **(G)** VF threshold in the two groups. *n* = 7 for each group. *p < 0.05. ^**^p < 0.01. ^***^p < 0.001. ^*⁣*⁣**^p < 0.0001. LSG, left stellate ganglion; LVB, left ventricular base; LVA, left ventricular apex; LVM, mid left ventricle; ERP, effective refractory period; APD, action potential duration; VAs, ventricular arrhythmias; VPBs, ventricular premature beats; nSVT, non-sustained VT; SVT, sustained ventricular tachycardia; VF, ventricular fibrillation.

First, we performed a left thoracotomy at the fourth intercostal space. Then we made an incision on the pericardium and occluded the left anterior descending coronary artery (LADO) below the first diagonal branch to establish a model of myocardial ischemia. Acute ischemia was confirmed by the alteration of ST segments and T-waves. Spontaneous VAs included ventricular premature beats (VPBs), non-sustained ventricular tachycardia (nSVT), sustained ventricular tachycardia (SVT), and ventricular fibrillation (VF). The patterns of VAs are shown in Figure 2C. VAs were defined according to the Lambeth Conventions (16) and numbered manually by experienced physicians.

### Histopathological staining

To know the efficiency of viral infection and Bmal1 knockdown, we analyzed the expression of mCherry (Abcam) by using double immunofluorescence staining with tyrosine hydroxylase (TH; Abcam). To further confirm the results of neural activity, we analyzed the expression of nerve growth factor (NGF; Abcam) and c-fos (Abcam) as two biomarkers of neural activity by double immunofluorescence staining with TH. In addition, 4,6-diamidino-2-phenylindole (DAPI) was stained to label the nucleus. The details of the experiments including tissue dissection, immunofluorescence staining, and data analysis were as previously described (17).

### RNA sequencing

At the end of our experiments, LSG tissue in each group was immediately collected, put into test tubes, and frozen in liquid nitrogen. Then, we selected high-quality samples (*n* = 3) to apply the following steps. The details of the experiments including RNA extraction, reverse transcription, and gene expression analysis were as previously described (14). After gene expression analysis, we conduct Kyoto Encyclopedia of Genes and Genomes (KEGG) classifications and KEGG pathway enrichment and Gene Ontology (GO) biological process annotations. The *p*-value was calculated and adjusted by the false discovery rate (FDR). Then, the adjusted *p*-value was renamed the Q value, and statistical significance was defined as a Q value ≤ 0.05.

### Quantitative RT–PCR

The tissue from the LSG was collected at the end of the experiment and immediately stored at -80°C in the refrigerator for subsequent analyses. The mRNA expression of Bmal1 was quantitatively analyzed by real-time PCR. The details of the operation were as previously described (1). The forward primer sequences of Bmal1 are “AAGGGAAGCTCACAGTCAGAT” and the reverse primer sequences are “GGACATTGCGTTGCATGTTGG.”

### Statistical analysis

All results are shown as the mean ± SEM. The Shapiro–Wilk normality test was used to test for a normal distribution. Data from the two groups were compared with an unpaired Student’s *t*-test. Fisher’s exact test was performed to analyze the incidence of SVT/VF in the two groups. GraphPad Prism software version 8.3.0 (GraphPad Software, La Jolla, California) was used to analyze all the data. Statistical significance was defined as a *p*-value of < 0.05.

## Results

### Efficiency evaluation of Bmal1 knockdown in the left stellate ganglion

Against sliced LSG, double immunofluorescence staining was performed for tyrosine hydroxylase (TH) and mCherry. TH was stained to label sympathetic neurons in the LSG. Meanwhile, mCherry was stained as the marker protein to show the areas where the virus was transfected (Figure 1D). A quantitative analysis of double immunofluorescence proved the high and equal efficiency of viral transfection in LSG sympathetic neurons in both the Bmal1 knockdown group and the control group (Figure 1E). We also used quantitative RT-PCR to verify that Bmal1 in the LSG was successfully knocked down compared with the control group (Figure 1F). All these results confirmed the Bmal1 in the LSG sympathetic neurons has been sufficiently knocked down by AAV.

### Bmal1 knockdown in the left stellate ganglion significantly stabilized left ventricular electrophysiological properties and reduced myocardial ischemia-induced VAs

We explored the effects of Bmal1 knockdown on left ventricular electrophysiological properties against canines. We found that ERP and APD90 at three sites (LVB, LVM, and LVA) were prolonged, which demonstrated that left ventricular electrophysiological properties were significantly stabilized (Figures 2A,B).

Furthermore, we established acute myocardial ischemia and analyzed ischemia-induced VAs, including VPBs, VT, and VF. The VPBs for 1 h were significantly reduced by Bmal1 knockdown (Figure 2D). Compared with the control group, the Bmal1 knockdown group exhibited fewer non-sustained VT (nSVT) as well (Figure 2E). The incidence of sustained VT/VF was also significantly decreased by Bmal1 knockdown (Figure 2F). Finally, we observed that Bmal1 knockdown significantly elevated the VF threshold which is an indicator to evaluate the ability to induce VF (Figure 2G). All these data suggested that Bmal1 knockdown in the LSG significantly stabilized left ventricular electrophysiological properties and reduced myocardial ischemia-induced VAs.

### Bmal1 knockdown in the left stellate ganglion altered the sympathetic tone, left stellate ganglion function, and the neural activity

**FIGURE 3:**
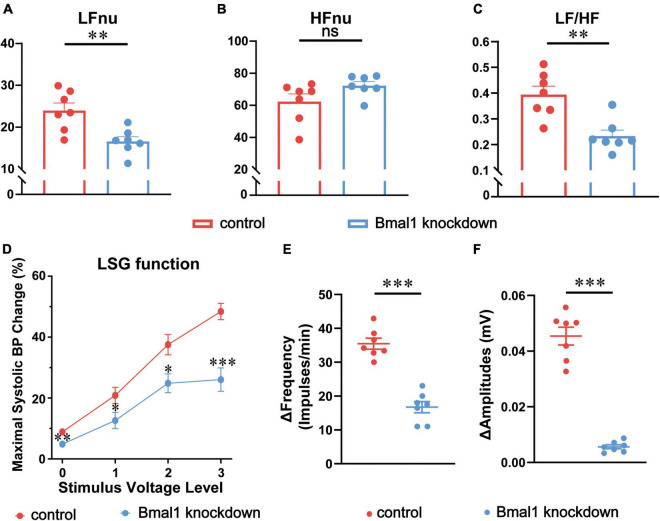
Effects of Bmal1 knockdown on the sympathetic tone, LSG function, and neural activity. The HRV indicators recorded by the Power Lab System: **(A)** LFnu, **(B)** HFnu, **(C)** LF/HF. **(D)** Maximal systolic BP change (LSG function) at 4 voltage levels. Alteration in the **(E)** frequency and **(F)** amplitude of LSG neural firing (LSG neural activity) in the two groups after MI. *n* = 7 for each group. *p < 0.05. ^**^p < 0.01. ^***^p < 0.001. LSG, left stellate ganglion; LFnu, normalized low-frequency power; HFnu, normalized high-frequency power; BP, blood pressure.

To explain the difference in cardiac electrophysiology in the two groups, we first analyzed the effect of Bmal1 knockdown on the sympathetic tone, HRV (LFnu, HFnu, and LF/HF) being an indicator of the sympathetic tone was recorded by the Power Lab System on the experiment day against canines. The amplitudes of LFnu (Figure 3A) and LF/HF (Figure 3C) were significantly attenuated by Bmal1 knockdown, which reflected the decline of the sympathetic tone. We also observed that HFnu was not altered by Bmal1 knockdown, indicating that the vagal tone was not affected (Figure 3B). Thus, Bmal1 knockdown in the LSG could remarkably attenuate the sympathetic tone.

Given that LSG is a part of the sympathetic nerve system, we began to focus on the electrophysiological properties of LSG. Neural function and neural activity are two main indicators to evaluate the electrophysiological properties of LSG. At the same time, the susceptibility of SNS to ischemia has been demonstrated to be the crucial reason for VAs after myocardial infarction (18) and our previous studies also observe the activation of LSG after acute myocardial infarction (14, 17). To assess LSG function, we observed maximal systolic BP changes after stimulating LSG, and to assess LSG neural activity, we also recorded neural discharging before myocardial ischemia (MI) and after MI against canines. LSG function was found to be significantly attenuated by Bmal1 knockdown at four different voltage levels of stimulation before MI (Figure 3D). Changes in the frequency and amplitude of LSG neural activity after MI were significantly attenuated by Bmal1 knockdown (Figures 3E,F). In general, these results indicated that Bmal1 knockdown in the LSG could inhibit LSG function and LSG neural activity.

### Bmal1 knockdown downregulated the expression of left stellate ganglion’s two markers c-fos and nerve growth factor

**FIGURE 4:**
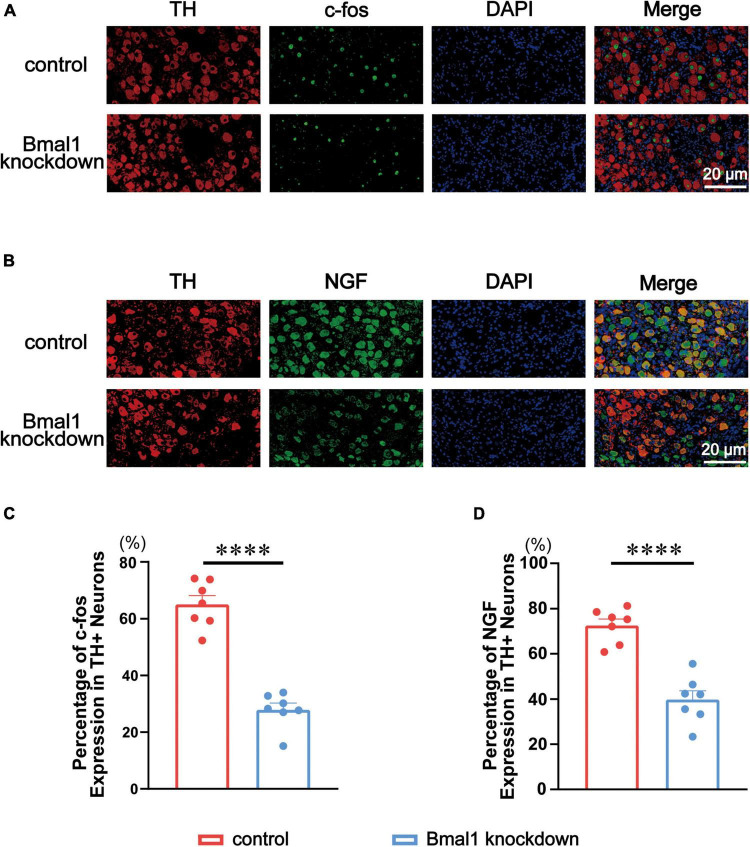
Effects of Bmal1 knockdown on c-fos/NGF expression in the LSG. **(A,B)** Representative immunofluorescent co-staining for c-fos/NGF with TH in the LSG in both groups. **(C,D)** Quantitative analysis of c-fos/NGF expressed as the percentage of TH + neurons. *n* = 7 for each group. ^***^p < 0.001. ^*⁣*⁣**^p < 0.0001. LSG, left stellate ganglion; NGF, nerve growth factor; TH, tyrosine hydroxylase.

c-fos and NGF are the classical markers of neural activity in the peripheral nervous system we used before (17). To know the expression of c-fos and NGF, we co-stained them with TH through double immunofluorescence in the control group and the Bmal1 knockdown group (Figures 4A,B). A quantitative analysis of double immunofluorescence showed that c-fos (Figure 4C) and NGF (Figure 4D) in the LSG were significantly decreased by Bmal1 knockdown compared with the control group. These results further confirmed that Bmal1 knockdown in the LSG was able to diminish the LSG neural activity.

### Transcriptome profiles of inhibitory effects on neural activity induced by Bmal1 knockdown in the left stellate ganglion

**FIGURE 5:**
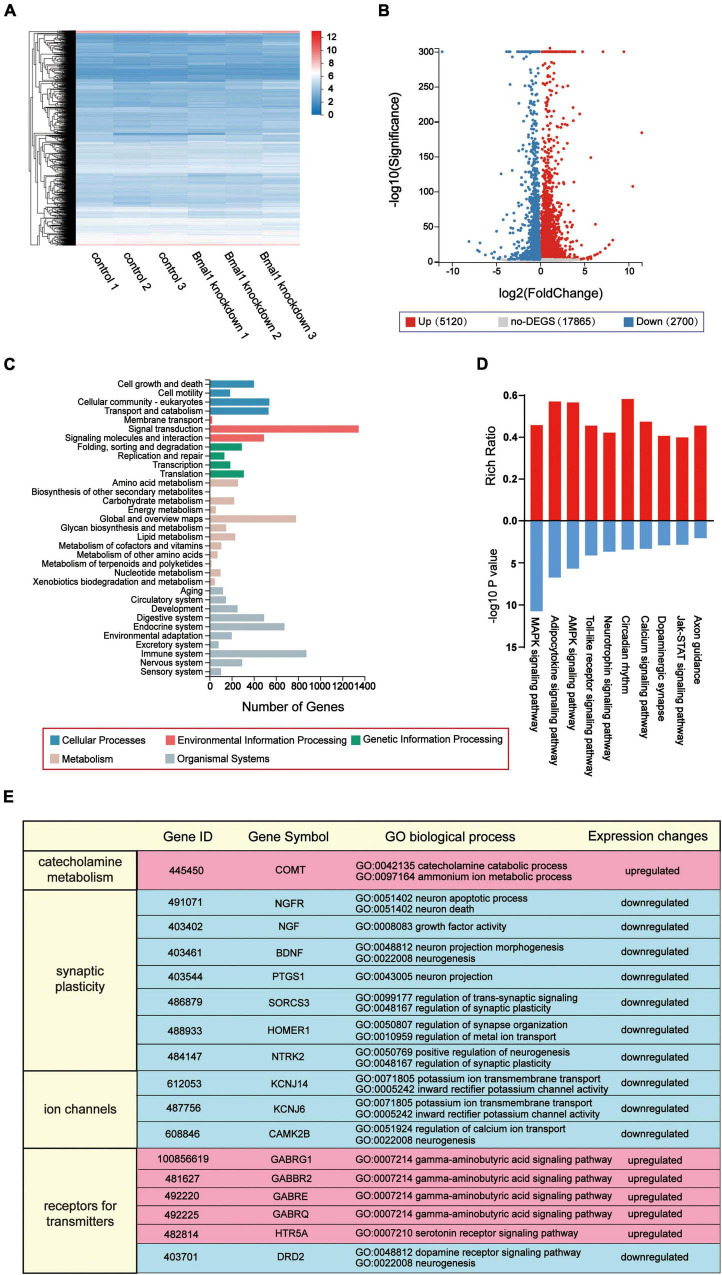
Transcriptome profiles of inhibitory effects on neural activity induced by Bmal1 knockdown in the LSG. **(A)** Heatmap showing 7,820 genes with altered expression induced by Bmal1 knockdown. **(B)** Volcano plot showing upregulated genes, downregulated genes, and not the differentially-expressed genes in the Bmal1 knockdown group compared with the control group. **(C)** KEGG classifications of differentially expressed genes induced by Bmal1 knockdown. **(D)** KEGG pathway enrichment analysis of DEGs induced by Bmal1. **(E)** Part of GO biological process annotations of differentially expressed genes associated with neural activity induced by Bmal1 knockdown. Differentially expressed genes were identified as methods were mentioned. *n* = 3 for each group. LSG, left stellate ganglion; DEGs, differentially expressed genes; KEGG, Kyoto Encyclopedia of Genes and Genomes; GO, Gene Ontology.

Bmal1 has been reported to be a transcription factor that regulates the expression of genes participating in various molecular and cellular processes as we have mentioned before. We predicted that its knockdown in the LSG caused global changes in the expression of genes involved in LSG neural activity and finally induced the difference in cardiac electrophysiology in the two groups. Thus, transcriptome profiles of these LSGs were analyzed by RNA sequencing, and differentially-expressed genes were identified. In our result, compared with the control group, 7,820 genes were significantly altered (5,120 genes were upregulated, 2,700 genes were downregulated) and 17,865 genes were not differentially expressed in the Bmal1 knockdown group (Figures 5A,B). Then, we analyzed the KEGG pathway classifications of differentially-expressed genes (Figure 5C). Most of them could be grouped into “signal transduction,” “immune system,” and “global and overview maps.” Through KEGG functional enrichment, we highlighted the top 10 KEGG pathways (Figure 5D). The results proved that Bmal1 knockdown broadly altered gene expression in various pathways in the LSG.

To explore the mechanisms for the decline in LSG neural activity induced by Bmal1 knockdown, we focused on the differentially-expressed genes in the nervous system according to the KEGG pathway classifications. A total of 176 genes were upregulated, and 119 genes were downregulated in the nervous system. Furthermore, we performed GO classifications against these differentially-expressed genes. According to GO annotation, we highlighted six upregulated genes, which were involved in the categories of catecholamine metabolism, synaptic plasticity, ion channels, and receptors for transmitters. These categories were mostly related to the LSG neural activity (Figure 5E). In summary, the transcriptome profiles demonstrated that the knockdown of transcription factor Bmal1 significantly altered the gene expression in the nervous system, which could serve as a molecular mechanism for the decline in LSG neural activity.

## Discussion

### Major findings

**FIGURE 6:**
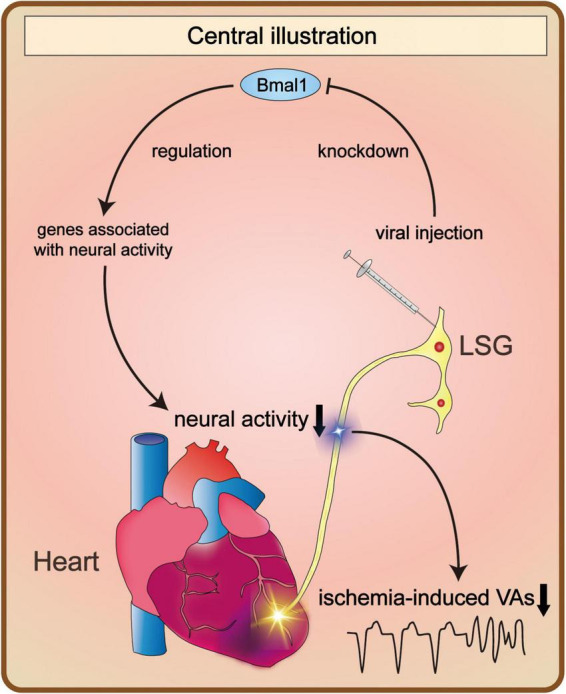
Bmal1 knockdown in the LSG altered its neural activity and the occurrence of VAs. Bmal1 knockdown led to changes in the transcriptional levels of genes associated with neural activity. As a result, it contributed to the alteration of LSG neural activity. Consequently, the occurrence of VAs induced by myocardial ischemia was obviously decreased. LSG, left stellate ganglion; VAs, ventricular arrhythmias.

In the present study, we demonstrated that Bmal1 knockdown in the LSG inhibited the function and neural activity of the LSG. Therefore, ventricular electrophysiological properties before MI were stabilized, and ischemia-induced VAs were suppressed by Bmal1 knockdown-mediated decline of LSG neural activity. Regarding the molecular mechanism, we demonstrated that Bmal1 regulated the transcription of genes associated with neural activity. These genes were significantly influenced by Bmal1 knockdown, contributing to changes in LSG function and neural activity (Figure 6).

### Bmal1 modulates the expression of genes associated with neural activity in the left stellate ganglion

Numerous experiments have focused on the function of Bmal1 in different organs or cells. Bmal1 is also an important clock gene that encodes transcription factors regulating the expression of various genes in the cardiovascular system and the central nervous system as we mentioned before. Therefore, Bmal1 in the sympathetic nervous system may also modulate its neural activity by regulating genes associated with neural activity. Genes associated with neural activity could be mostly classified into four categories: catecholamine metabolism, synaptic plasticity, ion channels, and receptors for transmitters. Thus, we highlighted genes in the four categories. In the results of RNA sequencing, differentially-expressed gene (significantly upregulated genes) involved in catecholamine metabolism by Bmal1 knockdown was catechol-O-methyl transferase (COMT). In a recent study, Klimina et al. reported that Bmal1 knockdown in the hypothalamus of rats led to a 2-fold decline of catecholamine DA and NE urinary concentrations after 1 month (11). Furthermore, Curtis et al. showed that global deficiency of Bmal1 altered the expression of aortic catechol-O-methyl transferase, an enzyme clearing catecholamines (19), these conclusions were similar to our results. The differentially-expressed genes (significantly downregulated genes) involved in synaptic plasticity by Bmal1 knockdown were BDNF, HOMER1, NGF, NGFR, NTRK2, PTGS1(COX-1), and SORCS3. Marti et al. reported that forced work could result in reduced phosphorylation of Bmal1 and reduced expression of the synaptic plasticity-related protein in the prefrontal cortex (20). Coria-Lucero et al. have figured out the presence of E-box elements of Bmal1 in the promoters of BDNF through bioinformatic analysis (21). Furthermore, Bmal1 has been proved to bind the Homer1 gene promoter, and it is necessary

for inducing the Homer1a gene following sleep deprivation (SD) stress in mice (22). Bmal1 knockout caused a decline of COX-1 expression in endothelial cells in mice (23). The differentially-expressed genes (significantly downregulated genes) involved in ion channels by Bmal1 knockdown were KCNJ14, KCNJ6, and CAMK2B. Bmal1 has been shown to modulate cardiac ion channel transcription by binding to the enhancer box (E-box) elements in the promoters of these genes or altering the expression of other transcription factors that regulate ion channel transcription (24). The differentially-expressed genes involved in receptors for transmitters upregulated by Bmal1 knockdown were GABBR2, GABRE, GABRG1, GABRQ, and HTR5A; and the genes downregulated were DRD2. Barca-Mayo et al. reported that deletion of Bmal1 in the astrocytes of the brain reduces GABA absorption and regulates GABA receptor signaling (13). Therefore, from these results, we predicted that alteration of LSG neural activity attributed to changes of these genes by Bmal1 knockdown and thereby prevented ventricular arrhythmias after myocardial ischemia.

### Limitations

There are still some limitations to our study. First, because of the limitation of the neural recording devices, we could record the neural activity of the LSG only under anesthesia. Second, as we only built a model of acute myocardial ischemia, there are still demands to investigate the influence of Bmal1 knockdown in a model of chronic myocardial ischemia, which will provide opportunities to observe the prognosis of MI and explore the mechanisms. As a result, such studies will have increased significance for our clinical practice. Third, we focused on cardiac electrophysiology in this study, so we did not observe the indicators of cardiac function.

## Conclusion

Bmal1 knockdown led to changes in transcriptional levels of genes associated with neural activity, thereby preventing ventricular arrhythmias after myocardial ischemia.

## Data availability statement

The data of RNA sequencing presented in this study are deposited in the Sequence Read Archive (SRA) repository in ncbi (www.ncbi.nlm.nih.gov), accession number: SUB11539576.

## Ethics statement

This animal study was reviewed and approved by the Animal Welfare and Ethics Committee of Renmin Hospital of Wuhan University. The protocol number is WDRM 20180507.

## Author contributions

ZY, ZHL (2nd author), and LJ designed the study and wrote the manuscript. SZ, LN, YYW, LZ, YHW, ZHL (9th author), and ZHL (10th author) collected laboratory data. XX, ZYL, YZ, HZ, RL, and CP performed the statistical analysis. LY and HJ edited manuscript. All authors contributed to the article and approved the submitted version.
